# Stigma related to contraceptive use and abortion in Kenya: scale development and validation

**DOI:** 10.1186/s12978-019-0799-1

**Published:** 2019-09-06

**Authors:** Marlene Makenzius, Grace McKinney, Monica Oguttu, Ulla Romild

**Affiliations:** 1 Department of Public Health Sciences, Global Health (IHCAR), Karolinska Institutet, Tomtebodavägen 18A, 171 77 Stockholm, Sweden; 2 College of Health Sciences, School of Nursing Sciences, University of Nairobi, Nairobi, Kenya; 3 Kisumu Medical Education Trust – KMET, Kisumu, Kenya; 4 The Public Health Agency of Sweden, Ostersund, Sweden

**Keywords:** Scale development, Validation, Social stigma, Adolescent pregnancy, Abortion, Contraception

## Abstract

**Background:**

Stigma related to abortion and contraceptive use is a serious public health threat for young people, and validated scales to measure this stigma are scarce. The purposes of the study were to validate a newly constructed scale to measure the stigma of contraceptive use and to adapt a scale to measure the stigma of abortion.

**Methods:**

A study nested in a cluster-randomised trial. In 2017, data was collected from 633 secondary school youths, in a semi-urban setting in western Kenya. A qualitative pre-phase (face-validity) were initially utilised to draft and validate a seven-item scale to capture contraceptive use stigma (CUS) and to adapt the Stigmatizing Attitudes, Beliefs and Actions (SABA) scale (18 items), which captures aspects of abortion stigma. Statistical tests used included test-retest reliability analysis, Pearson’s correlation coefficients, Wilcoxon signed-rank test, Factor Analysis, Principal Component Analysis, interclass correlation and Cronbach’s alpha.

**Results:**

For the CUS scale, paired t-test and Wilcoxon signed-rank test showed no significant score changed between time points (*p* = 0.64; 0.67). CUS had similar patterns between time points, with two relevant components: *promiscuity* and *lack of autonomy*. Cronbach’s alpha indicated acceptable internal consistency between time points (0.71;0.7). The confirmatory factor loadings for each item in the modified three subscales of SABA had a similar pattern to the original SABA scale, in particularly regarding negative stereotyping and, excluding and discriminating factors. The Cronbach’s alpha was adequate, although lower for the modified SABA (0.74) as compared to the original SABA (0.9). The SABA scale was renamed into Adolescents Stigmatizing Attitudes, Beliefs and Action (ASABA) scale.

**Conclusions:**

The CUS scale is considered valid and reliable for measuring contraceptive use stigma, and the ASABA scale was rated as reliable for capturing abortion stigma based on negative stereotyping and excluding and discriminating factors. The CUS, up to date the first ever proposed CUS scale, and the ASABA scale can be used to measure effects of stigma reduction interventions with the aim of preventing unintended pregnancies, motherhood and unsafe abortion among adolescents in Kenya and similar low-resource settings.

## Plain English summary

Contraceptive use stigma and abortion stigma is acknowledged worldwide, but still poorly researched. Contraceptive use stigma and abortion stigma largely nourishes from gender stereotypes, which are used to deny girls and women access to abortion and contraceptive services. In particularly the stereotype ascribing women to the role of motherhood. This stereotype implies that women should prioritize childbearing and child caring over all other roles, even schooling. As a result (which in some cases are exacerbated by cultural and religious fundamentalism), there is a negative effect over the way a given society perceives abortion, as well as those who seek or have had an abortion, those who work in abortion care, and those who actively support abortion rights. Finally, contraceptive use stigma and abortion stigma serves to erase and disguise legitimate health services, discredit those who would provide or procure it and undermine those who advocate for its legality and accessibility. The roots of such stigma that intend to control female sexuality are based on social constructs that can be deconstructed. However, as in the case of HIV and AIDS, we must empirically determine what role stigma plays and how it is expressed in particular communities before we can effectively address the problem. This paper in the issue of *Reproductive Health* provides validated scales measuring contraceptive use stigma (CUS) and abortion stigma (ASABA), among adolescents. Adolescents are the far most affected population suffering from contraceptive use stigma and abortion stigma. The CUS scale, up to date the first ever proposed CUS scale and the ASABA scale can be used to measure effects of stigma reduction interventions with the aim of preventing unintended pregnancies, motherhood and unsafe abortion among adolescents in Kenya and similar low-resource settings.

## Background

Although access to safe abortions and effective contraceptives has increased worldwide, accessibility is still a great barrier in many low- and middle-income countries (LMIC) settings, including Kenya [1–5]. Social norms and attitudes related to contraceptive use and abortion continues to pose a serious threat to women’s health and quality of life across the world [6–8]. The risk of death associated with pregnancy is about a third higher among 15- to 19-year olds as compared to women of other ages [9–11].

Adolescents in LMICs, particularly unmarried ones, face a number of barriers in obtaining contraception and using them correctly and consistently [2, 3, 9, 12, 13]. One of these barriers is a strong taboo against sexual activity outside of marriage [12, 14–16], which makes it more difficult to collect and assess unmet-need data among this group. Surveys that ask about sexual activity do not always include unmarried women, and even when they do, these women may underreport their sexual activity and contraceptive use due to an unwillingness to risk social disapproval and stigmatising attitudes associated with nonmarital sexual activities [17–19]. In addition, both women and men have concerns about the effects of contraceptive methods on women’s bodies, including their weight, menstrual cycles, libido, sexual desirability and pleasure [20–23]. Such studies reveal that both men’s and women’s resistance to contraception could be related to traditional gender norms and power, or to a suspicion that outsiders (Westerners) aim to control women’s fertility [19].

Overall, sexually active unmarried women who are not using contraception make up an estimated 18% of all women in developing countries with unmet needs for any contraception method [13], and the proportion is estimated to be even higher among sexually active unmarried adolescents [19, 24, 25]. The majority of abortions result from unintended pregnancies (e.g., mistimed or unwanted pregnancies). Abortions occur as frequently in the most-restrictive category of country as they do in the least-restrictive category, 37 and 34 per 1000 women, respectively [8]. Worldwide, about 45% of all abortions are performed with unsafe procedures (e.g., by an individual who does not have the necessary training or in an environment that does not conform to minimal medical standards). Overwhelmingly, unsafe abortions occur in developing regions where restrictive abortion laws are concentrated, so as the case in Kenya [7, 8]. Abortion is not permitted in Kenya, unless, in the opinion of a trained health professional, there is need for emergency treatment, or the life or health of the mother is in danger. No safe abortion is offered in public health facility in Kenya. Safe abortion services can only be found in a few private hospitals at a higher cost and has frequently become the privilege of the rich. Abortion is still spoken to by the 1970 Penal Code which criminalises it, and the 2010 Constitution which makes exceptions to this criminalisation. The lack of clarity and transparency with regard to the circumstances in which abortion is legal greatly contributes to Kenya’s high maternal mortality ratio from complications of unsafe abortion [6–10, 24]. Meeting the unmet need of modern contraception for women aged 15–19 would reduce unintended pregnancies among this age group by 6 million annually. This could prevent 2.1 million unplanned births, 3.2 million abortions (most of them unsafe) and 5600 maternal deaths [13]. Despite the fact that the age of sexual consent in Kenya is 18 years, adolescent pregnancy is one of the major causes of school dropout among girls between 16 and 18 years of age [24], as well as a major cause of death among adolescent girls [9, 10, 26]. Sexual and reproductive health services in Kenya fall short of meeting adolescents’ needs. For example, an estimated 665,000 young women aged 15–19 in Kenya are married or sexually active and want to avoid becoming pregnant in the next 2 years [24]. However, increased knowledge on contraception may not be the only solution since high level of knowledge on contraceptives does not always amount to practice [27]. Contraceptive use among adolescents is sometimes associated with immorality and a promiscuous lifestyle, and the use is considered physically harmful [16, 17, 19, 20]. Such stigmatising attitudes may shame and silence young women about their contraceptive needs, which may lead to unintended pregnancies and unsafe abortions [19].

The social stigma surrounding unintended pregnancies and abortion plays a critical role in the social, medical and legal marginalisation of contraceptive services and abortion care [6, 28]. Historically, stigma theory has progressed from an individualistic focus towards the recognition of stigma as a socially constructed process [29]. Conceptualisations of sexuality and stigma are shaped at different levels within a society and are framed by a multiplicity of factors, including political structures and sociocultural context and intersections with other identities such as gender, socioeconomic status, race, ethnicity, religion and rural/urban location [30].

In 2013, Shellenberg et al. [31] developed a Stigmatizing Attitudes, Beliefs and Action (SABA) scale to measure stigmatising attitudes related to abortion. This framework referred to interrelated stigma components such as labelling, stereotyping, separating/excluding and discriminating [30]. The SABA scale was tested among adults in Ghana and Zambia [31]. The International Planned Parenthood Federation has adapted the SABA scale to the situation of young people, but it is not yet validated. To the best of our knowledge, at the beginning of this research, no validated scale or quantitative instrument specifically designed to measure CUS exists. If we can further understand and measure sociocultural stigma related to contraceptive use and abortion among the most affected population (adolescents), we will be better equipped to develop interventions to reduce such stigma.

This study aimed to develop and validate a scale to measure CUS and to validate SABAs among adolescents in western Kenya.

## Methods

### Study design and setting

This was a study, nested in a larger cluster randomised intervention trial (ClinicalTrial.gov NCT03065842), among secondary school youths in Kisumu, Kenya. Kisumu is the third-largest city in Kenya with a population of approximately 500,000 and bordering Lake Victoria. The overarching objective of the intervention trial was to reduce the stigma of adolescent girls using contraceptives and/or undergoing abortion procedures. For the current study the aim was to draft and validate instruments used in the trial to measure the effect of the intervention given to the students (evidence and rights based information, and value clarifications activities on abortion and contraceptive use). The project was conducted in collaboration between Karolinska Institutet in Sweden and Kisumu Medical and Education Trust (KMET), and in partnership with the Ministry of Education and the Ministry of Health in Kisumu County, located in the western region of Kenya. KMET is a nongovernmental organisation registered under the Trustee Act of 1995. KMET has no political, religious or governmental affiliation.

For the larger trial, where the current study was nested in, one intervention school and one control school were cluster-randomised from the regional sample frame of four schools. Inclusion criteria for the schools were as follows: semi-urban secondary day schools, mixed gender, with a minimum of 400 students. This study was based on data derived from the control school, which allowed data analysis without the intervention effect.

The population of Kisumu County consists primarily of the Luo and Kisii ethnic groups, mainly Christians who speak Luo, Kiswahili and English, like Kenya overall (83% Christians, 11.2% Muslims) [32]. The proportion of young people in Kisumu County is higher than the country average. That is, one in four (25%) people in Kisumu is an adolescent aged 10–19. A young population has implications for the county’s health and development agenda due to the demands put on the provision of services, including health and education. In Kenya, a main area of concern is the sexual and reproductive health of adolescents and the extent to which their sexual and reproductive health and rights (SRHR) are met. Half of the women aged 20–49 and men aged 20–54 first had sex by age 16 and 18, respectively. Sexual education is an important determinant for SRHR. Girls who complete secondary school are less likely to have an unintended pregnancy and are more likely to have higher socioeconomic status. About 95% of them are enrolled in primary school, but only 61% transition to secondary school, which is about the same as the national statistics [32].

### Face validity

Pre-phase work, based on a multi-method approach that was seen in the study of Shellenberg et al. [31], was conducted from November 2016 to January 2017. The procedure followed guidelines by the International Network for the Reduction of Abortion Discrimination and Stigma (Inroads) on how to adapt a scale to measure abortion stigma [33]. Participants in the pre-phase work were healthcare providers, teachers and students were invited to participate by a letter from a research assistant at KMET. Inclusion criteria’s were health care staff working at youth friendly clinics (*n* = 30) [16], teachers and adolescents from the school catchment-area. The pre-phase work aimed to discuss participants’ views on unintended pregnancy, abortion and contraceptive use among adolescents, and explore the construction of a scale to measure CUS and to explore the applicability of the SABA scale [31] in regards to adolescents in Kisumu. A total of four workshops and six focus group discussions were conducted, digitally recorded and verbally transcribed. They involved healthcare providers (*n* = 30), teachers (*n* = 19) and students (*n* = 21) to explore attitudes and beliefs associated with contraceptive use and abortion among adolescent girls.

Based on findings from the pre-phase work, a seven-item CUS scale was developed—it followed a standard procedure for Likert scale development, per DeVellis [34] (five-point Liker scale: 1 = strongly disagree, 2 = disagree, 3 = unsure, 4 = agree and 5 = strongly agree). The CUS scale captured stigmatising attitudes and beliefs regarding contraceptive use among adolescent girls. The applicability and clarity of the SABA scale was confirmed, but modified. The SABA scale captured three important dimensions of stigma related to women associated with abortion (18 items): Subscale 1, negative stereotypes (8 items); Subscale 2, discrimination and exclusion (seven items); and Subscale 3, potential contagion (three items) [31]. The format of the SABA scale was identical to the CUS scale that used a five-point Likert scale. The word *woman* was changed to *girl* in all questions, and two items were added to the scale. Further editing was done. For example, the true-or-false question (Q) *A married woman is more deserving an abortion than an unmarried woman* was added to Subscale 1, and Q6, *A woman who has had an abortion might encourage other women to get abortions*, was changed to *A girl who has had an abortion might be a bad influence on other women* and removed from Subscale 1 and added to Subscale 3. For Subscale 2, *A girl who has had an abortion should be prohibited from going to school* was added. The adapted SABA was renamed Adolescents’ Stigmatizing Attitudes, Beliefs and Actions (ASABA) scale. The CUS and ASABA scales were pretested (633 adolescents) as a baseline survey, i.e. time point (TP) I and were followed up with a repeat test of 548 of the 633 adolescents after 1 month (TP II).

### Statistical methods

The scales of CUS and ASABA were statistically tested. According to DeVellis [34], there are variations in suggested sample sizes needed for scale development, but a minimum of five respondents per scale item is recommended, which the current sample of 633 individuals exceeded. The reliability of the CUS scale was tested by using paired sample t-tests and a Wilcoxon signed-rank test to compare total sum scores, means (SD), and mean ranks, and for each item between time points. In addition, Kaiser-Meyer-Olkin (KMO) was performed for each survey at each of the two time points in order to verify that the data was suitable for Principle Component Analysis (PCA) [35]. Exploratory Factor Analaysis (FA) with PCA is the standard procedure to validate new scales [36], ,and the result from TP I was compared to TP II. As there was no previous structure to follow, components with eigenvalues > 1.0 were included, and questions that had a factor loading ≥0.40 were retained. Oblique (promax) rotation was used, with a kappa of 4, to help create a simple structure that contained interpretable factors and to allow for correlation between the items. Cronbach’s alpha was used to measure internal consistency and reliability of a psychometric [37, 38].

The statistical analysis for the ASABAs was based on the previous validation of the SABA scale in Ghana and Zambia by Shellenberg et al. [31] For that aim, a parallel-form reliability analysis was conducted to compare the results of the ASABA scale to the results of the SABA scale that was validated among heterogeneous groups from Ghana and Zambia in 2013 [31]. A confirmatory PCA analysis was used to compare responses for the total scale and for each subscale (component) [36]. The comparisons were further analysed by using Cronbach’s alpha coefficients, which provided evidence of internal consistency and reliability, given that all four coefficients surpassed the minimum standard for reliability (0.60). KMO, PCA, Wilcoxon signed-rank tests and Cronbach’s alpha, medians (SD) were generated with SPSS (Version 23).

## Results

The cluster-randomised study comprised in total 1207 students, distributed between one intervention school (*n* = 574) and one control school (*n* = 633), both Christian secondary schools located in semi-urban areas in western Kenya. To avoid the intervention effect between time points, the analysis in the current study was based on the 633 students from the control school. Out of the 633 students, 325 (51.3%) were females, 305 (48.2%) were males, and 3 (0.5%) students did not reveal their sex. The mean age was 16.8 years (16.7 for females and 17.00 years for males).

The reliability of the CUS scale between TP I and TP II are shown in Table 1. The results shows that Q2 has a mean score of 4 at TP I but a mean score of 3.09 for TP II. In Q7, the mean score was 2.10 for TP I, compared to 1.86 for TP II. As for Questions 1, 3, 4, 5 and 6, the mean scores are stable between time points. The paired t-test (used as a test-retest measure) of the total score was not significant (mean difference of 0.13, *p* = 0.64), suggesting no total score-generating mechanism changes from TP I to TP II. Wilcoxon signed-rank test on total score, confirmed the paired t-test, with a *p*-value of 0.67.

**Table 1:** Mean (SD) score for each question of the CUS at TP I (*n* = 608) and TP II (*n* = 517)

Question Score 1–5		Time Point I n = 608^a^ Mean (SD)	Time Point II n = 517^a^ Mean (SD)	Wilcoxon signed-rank test
1	A girl who uses a contraceptive method is promiscuous (sexually immoral, likes to have many sexual relationships).	3.10 (1.37)	3.00 (1.33)	0.061
2	A girl who use a contraceptive method will encourage other girls to have a promiscuous lifestyle.	4.00 (1.29)	3.09 (1.31)	0.002
3	A girl cannot decide for herself to use a contraceptive method.	2.37 (1.27)	2.50 (1.29)	0.052
4	A married woman is more deserving of a contraceptive method than an unmarried woman.	2.68 (1.38)	2.63 (1.35)	0.392
5	A girl who uses contraceptives will have problems when she decides to get pregnant.	3.15 (1.45)	3.07 (1.37)	0.457
6	A girl who carries condoms is likely to have many sexual partners.	2.88 (1.48)	2.84 (1.44)	0.212
7	A girl should not insist on using a condom; it is the man to decide whether to use a condom.	(1.23)	2.10 (1.28)	< 0.001

^a^Only those who answered all questions were included in the analysis. Internal dropout rate on specific items was 1.3–2.1% (*n* = 8–13)

The CUS data was found appropriate for PCA analysis, as its KMO values were 0.68 for TP I and 0.69 for TP II. As illustrated in Table 2, two components were retained under orthogonal (promax) rotation for both time points. All questions of the CUS scale have or are close to having factor loadings ≥0.40 for both time points of one component. For Component 1, Questions 1, 2, 5 and 6 all had factor loading values ≥0.40 at TP I and II. Questions 3 and 7 load in TP II of Component 1 and TP I of Component 2. The internal consistency for the CUS was 0.713 at TP I and 0.697 for TP II.

**Table 2:** Results of the PCA for the CUS scale at each time point with oblique (promax) rotation TP I (n = 608) and TP II (n = 517)

Question	Component 1		Component 2	
	Time Point I	Time Point II	Time Point I	Time Point II
1	**0.825**	**0.748**	−0.206	− 0.039
2	**0.791**	**0.825**	−0.074	−0.186
3	−0.132	0.072	**0.682**	**0.702**
4	0.165	**0.402**	**0.415**	0.276
5	**0.545**	**0.544**	0.290	0.095
6	**0.434**	**0.434**	0.398	0.288
7	−0.093	−0.095	**0.725**	**0.850**

Bolded factor loadings ≥0.40

The SABA scale was initially adapted, and the added Q, *A married woman is more deserving of an abortion than an unmarried woman* had a mean of 2.04 (SD = 1.23). The Q *A girl who has had an abortion should be prohibited from going to school* had a mean of 1.41 (SD = 0.81), and Q6 in Subscale 1 was moved to Subscale 3. The analysis revealed that when the Q *A married woman is more deserving of an abortion than an unmarried woman* was added to Subscale 1, the KMO decreased from 0.76 to 0.74 and the factor loading was < 0.40 (0.14). Similarly, when adding the Q *A girl who has had an abortion should be prohibited from going to school* to Subscale 2, the KMO slightly decreased from 0.78 to 0.77. When Q6 was removed from Subscale 1 and added to Subscale 3, the KMO increased from 0.56 to 0.63, but the total variance explained in Subscale 3 decreased from 44.60 to 37.92. Consequently, the structure of the original SABA was retained for the ASABA scale and revealed a KMO of 0.815, with 48.96% explained variance in the data for the full 18-item scale.

Table 3 shows similar patterns between ASABA and SABA regarding the mean score of subscales and total scores. Overall, the SD for the three subscales of SABA shows a larger variation in the data compared to the ASABA scale (*p* <  0.001). Consequently, the scores of the ASABA scale are closer to the mean, compared to the result of the SABA scale (Table 3).

**Table 3:** Stigmatising attitudes, beliefs and actions among secondary students in Kenya (*n* = 633) as compared to the heterogeneous sample from Ghana and Zambia (*n* = 531)

Scale items ASABA scale (SABA scale)		Kenya (*n* = 613) Subscale and item score Mean (SD)	Ghana and Zambia^a^ (n = 531) Subscale score Mean (SD)
Stigmatising attitudes, 8 items (min-max = 8–40)		28.03 (5.76)	25.7 (7.48)
1	A girl who has an induced abortion is committing a sin.	4.49 (1.06)	
	(A woman who has an abortion is committing a sin.)		
2	Once a girl has one abortion, she will make it a habit.	3.38 (1.31)	
	(Once a woman has one abortion, she will make it a habit.)		
3	A girl who has an abortion cannot be trusted.	3.05 (1.42)	
	(A woman who has had an abortion cannot be trusted.)		
4	A girl who has an abortion brings shame to her family.	3.92 (1.23)	
	(A woman who has had an abortion brings shame to her family.)		
5	The health of a girl who has an abortion is never as good as it was before the abortion.	3.78 (1.36)	
	(The health of a woman who has had an abortion is never as good as it was before the abortion.)		
6	A girl who has had an abortion might be a bad influence on other women.	3.29 (1.41)	
	(A woman who has had an abortion might encourage other women to get abortions.)		
7	A girl who has an abortion will be a bad mother.	2.62 (1.36)	
	(A woman who has an abortion is a bad mother.)		
8	A girl who has an abortion brings shame to her community.	3.46 (1.36)	
	(A woman who has an abortion brings shame to her community.)		
Exclusion and discrimination, 8 items (min-max = 8–40)		14.31 (4.66)	15.72 (5.79)
9	A girl who has had an abortion should be prohibited from going to religious services. (A woman who has had an abortion should be prohibited from going to religious services.)	1.54 (0.88)	
10	A girl who has had an abortion should be teased so that she will be ashamed about her decision.	2.14 (1.29)	
	(I would tease a woman who has had an abortion so that she will be ashamed about her decision.)		
11	A girl should be disgraced in my community if she has had an abortion.	2.09 (1.17)	
	(I would try to disgrace a woman in my community if I found out she had an abortion.)		
12	A man should not marry a woman who has had an abortion.	2.06 (1.16)	
	(A man should not marry a woman who has had an abortion because she may not be able to bear children.)		
13	A girl who has had an abortion should no longer be associated with me.	1.90 (1.12)	
	(I would stop being friends with someone if I found out that she had an abortion.)		
14	A girl who had an abortion should be pointed fingers at so that other people would know what she has done.	1.81 (1.12)	
	(I would point my fingers at a woman who had an abortion so that other people would know what she has done.)		
15	A girl who has an abortion should not be treated the same as everyone else.	1.75 (1.04)	
	(A woman who has an abortion should not be treated the same as everyone else.)		
Fear of contagion, 3 items (min-max = 3–15)		5.86 (2.19)	7.54 (3.41)
16	A girl who has had an abortion can make other people fall ill or get sick.	1.68 (1.04)	
	(A woman who has an abortion can make other people fall ill or get sick.)		
17	A girl who has had an abortion should be isolated from other people in the community for at least 4 weeks after having an abortion.	2.20 (1.12)	
	(A woman who has an abortion should be isolated from other people in the community for at least 1 month after having an abortion.)		
18	If a boy has sex with a girl who has had an abortion, he will most likely become infected with a disease.	1.96 (1.11)	
	(If a man has sex with a woman who has had an abortion, he will become infected with a disease.)		
Total scores (min-max = 18–85)		47.19 (9.27)	48.9 (14.2)

^a^Internal dropout on specific items 0.0–1.7%

Table 4 shows confirmatory factor loadings for each and one of the three subscales of ASABA in relation to the SABA scale. The three-factor structure of the ASABA scale, negative stereotyping (KMO 0.76), exclusion and discrimination (KMO 0.78) and fear of contagion (KMO 0.56), explained variance in the data with 43.34, 35.89 and 44.6%, respectively. The explained variance was 49% for the total 18-item scale (KMO 0.82). As shown in Table 4, Q1 loaded < 0.40. However, Q1 loaded stronger in a fourth component (0.9).

**Table 4:** Results of confirmatory analysis of the ASABAs (n = 613), analysed with principal component analysis with direct oblique rotation, among secondary students in Kenya (n = 633), compared to the heterogeneous sample from Ghana and Zambia (n = 531)

Question	Subscale 1		Subscale 2		Subscale 3	
	ASABA	SABA	ASABA	SABA	ASABA	SABA
1	.202^a^	.750	–	–	–	–
2	.448	.630	–	–	–	–
3	.669	.720	–	–	–	–
4	.680	.480	–	–	–	–
5	.539	.610	–	–	–	–
6	.452	.460	–	–	–	–
7	.628	.740	–	–	–	–
8	.631	.670	–	–	–	–
9	–	–	.467	.610	–	–
10	–	–	.560	.660	–	–
11	–	–	.663	.530	–	–
12	–	–	.601	.820	–	–
13	–	–	.646	.620	–	–
14	–	–	.676	.580	–	–
15	–	–	.553	.640	–	–
16	–	–	–	–	.600	.870
17	–	–	–	–	.656	.590
18	–	–	–	–	.740	.870

^a^Loaded 0.9 in fourth component

Table 5 presents the interclass correlations for the subscales and total scale scores for the full sample, showing significant correlations (*p* <  0.001–0.001).

**Table 5:** Interclass correlations for the three subscales and the full ASABA scale

Subscales	Negative stereotypes (n = 613)	Exclusion and discrimination (*n* = 603)	Fear of contagion (*n* = 612)	Total ASABA (*n* = 602)
Negative stereotypes	1	.231 < .001^a^	.138.001^a^	.772 < .001^a^
Exclusion and discrimination	.231 < .001^a^	1	.477 < .001^a^	.764 < .001^a^
Fear of contagion	.138.001^a^	.477 < .001^a^	1	.566 < .001^a^
Total ASABA	.772 < .001^a^	.764 < .001^a^	.566 < .001^a^	1 < .001^a^

^a^ Correlation significant at .05 level

The internal consistency coefficients for the ASABA scale compared to the SABA scale are lower for all three subscales. The ASABA subscales of negative stereotyping, exclusion and discrimination had coefficients like the ones of the SABA scale. However, the ASABA subscale of fear of contagion had a lower internal consistency coefficient than that of the SABA scale (0.38 compared to 0.80). The overall coefficient was lower for the ASABA (0.74) as compared to the SABA (0.9) (Table 6).

**Table 6:** Internal consistency coefficients for ASABA, compared to SABA (*n* = 613)

Subscales	Set of items	Cronbach’s alpha
	ASABA (SABA)	ASABA (SABA)
Negative stereotyping of women	8 (8)	0.67 (0.85)
Exclusion and discrimination	7 (7)	0.70 (0.8)
Fear of contagion	3 (3)	0.38 (0.8)
Total items	18 (18)	0.74 (0.9)

## Discussion

The aims of this study were to develop a scale to measure CUS and to adapt and validate a scale to measure abortion stigma among adolescents. The findings strongly indicates that CUS scale is reliable to measure stigma related to contraceptive use among adolescent girls, The ASABA scale was rated reliable to capture abortion stigma based on negative stereotyping and excluding and discriminating factors. Both scales are timely relevant since the potential social, economic and health-related consequences of unintended pregnancies among adolescent girls [6, 9–11, 13] makes it essential to measure and understand unmet needs for contraception and safe abortions. Although the Kenyan Constitution offers potential for increasing women’s access to safe abortion, societal attitudes towards young women who procure abortion remain largely negative. While stigma, and access barriers to safe abortion and modern contraceptives continues to be neglected, young women will not only delay the request for termination but also use resort to unsafe and life threaten options.

The two components of the CUS were labelled *promiscuity* and *lack of autonomy*. These labels were chosen based on the themes shared by the questions contained in each component. Most of the questions that loaded in Component 1 share the theme of contraceptive use being associated with promiscuity, which can be seen either in the characteristics of girls who use contraceptives or in the belief that girls who use contraceptives encourage others to be promiscuous. However, Q5, *A girl who uses contraceptives will have problems when she decides to get pregnant*, does not fit this label as well as the other questions. At face value, this question may not relate to promiscuity, as it proposes that contraceptive use will result in fertility issues later in life. However, the issue can be looked at from another angle. *A girl who uses a contraceptive method is promiscuous* claims that a girl who uses contraceptives is inherently promiscuous. Taking this into account, if girls who use contraceptives are promiscuous and are therefore at greater risk for catching STIs, this would impact their ability to have children later in life [39].

Component 2 was labelled *lack of autonomy*, suggesting that a girl is not supposed to, or should not be allowed to, make the decision to use contraception, and that this responsibility should be put on someone else (for example, her partner or her parents). The distribution of power in the developing world (and particularly in sub-Saharan Africa) is unequal, and men are often the primary decision makers about family size and the use of family planning.

Some differences in the results between the scales of ASABA and SABA may be explained by different sociodemographic samples. The original SABA scale was tested on a heterogeneous sample, while the ASABA scale was tested on a homogenous sample. The SABA scale was tested in Ghana and Zambia among participants with different background characteristics [31], whereas the sample that tested the ASABA scale came only from the Kisumu region of Kenya. Specifically, the respondents for the ASABA scale all came from a Christian secondary school. Moreover, the age range for the sample of SABA scale was larger, and the original sample included individuals aged 18 to 54 [31], while the sample of the current ASABA scale included students aged 13 to 21. People of different ages tend to have different views about social issues because of where they are in their lives and their level of openness [40]. Also, older generations tend to be more conservative regarding social issues as compared to younger generations [41]. The larger variety in age, as well as the higher mean age, in the SABA sample could therefore have yielded different results than the younger ASABA sample.

Overall, the similarities in loadings in SABA and ASABA were meant to retain the three components (subscales) from the original SABA on the ASABA. These were negative stereotyping, exclusion and discrimination and fear of contagion. The adapted version did not seem to add relevant information that could strengthen the tool. Most items on ASABA loaded similar to what they loaded on the SABA, despite their adaption to a new population, with one exception. First, the item in Subscale 1: *A girl who has had an abortion is committing a sin*, seems to have an overarching position, as it loaded 0.9 in the fourth component. Most students held the opinion that abortion is committing a sin, regardless if their overall opinions were not stigmatising. Most people in Kenya are highly religious, and teaching in schools is faith-based. The applicable school was a Christian school, and its teaching is based on Christian principles and values, which may explain this item’s overarching role in the scale. Given this, the underlying themes and the subscales they define are considered to be the same as the SABA scale. However, the use of ASABA should provide the option to exclude the third subscale, fear of contagion, since this subscale had a notable lower internal consistency compared to the result found in the sample of Ghana and Zambia [31].

One limitation in comparing the ASABA to the SABA is the unclear validation procedure of Shellenberg et al. [31] Shellenberg et al. explains that the data from one time point was explored by using an exploratory FA and then a PCA by using promax rotation, but the description of how components were retained is vague [31]. Part of how they decided to retain components was by way of ‘interpretability factors’ [31]. These factors were likely judgement calls that cannot be replicated. For this reason, parallel analysis was used for comparison and may not have been analysed in a comparable way to the original SABA validation study. This, however, is clearly not a direct limitation of our data. It simply limited our ability to fairly compare with the results of Shellenberg et al. Additionally, although the test-retest is a strength, the follow-up time of 1 month could be considered too long. The length of time between the first testing and the retest is critical [41]. A long follow-up time allows for true changes in attitude [41]. Secondary students may also be impressionable, and their views change frequently as they gain life experience (including gaining an education) or interactions with family and friends. This could have influenced the measure of the stability of the scales. A final limitation was the presence of missing data, which may account for some of the differences between time points for CUS. For both time points, not all the participants answered all the questions. Although the missing values were considered to be by random chance and not systemic in nature, participants with missing data were excluded in the paired sample analysis to minimise their effect. As a result, data for some individuals was not used in the analysis, which caused the sample size to be less than the total number of participants and which could have had an impact on the findings.

The CUS scale and the ASABA scale may be useful to measure societal attitudes and cultural beliefs around gender roles related to SRHR. The information can be used in the design of behaviour change campaigns and comprehensive sexual education, in the development and introduction of new contraceptive methods, and in provider training and service delivery, including counselling about methods. In the long run, this may increase female empowerment at community and household levels for a sustainable equality movement regarding SRHR.

## Conclusion

The CUS scale is considered validated and reliable to measure contraceptive use, based on attitudes that suggest that girls who use contraceptives are potentially promiscuous and that girls lack autonomy to decide whether to use contraceptives. The ASABA scale is rated reliable to capture abortion stigma based on negative stereotyping and excluding and discriminating factors. However, further validations may be needed to investigate the potential impact that fear of contagion may have on abortion stigma. The CUS and ASABA scales can be used to measure effects of stigma reduction interventions with the aim to prevent unintended pregnancy, motherhood and unsafe abortion among adolescents in Kenya and similar low-resource settings.

## Data Availability

Data is yet to be analysed for other publications, hence the availability of data is currently limited. Contact marlene.makenzius@ki.se to request data availability.
